# A Pilot Placebo‐Controlled Randomized Trial of Repeated High‐Dose Intravenous Magnesium Sulphate and High‐Dose Nebulized Budesonide for Severe Asthma in the Paediatric Emergency Department

**DOI:** 10.1111/jpc.70530

**Published:** 2026-08-16

**Authors:** Khalid Alansari, Fatin Abou Moussa, Hadi Abu Rasheed, Bruce L. Davidson, Khalid Yousif Ibrahim, Mahmoud Alrifaai

**Affiliations:** ^1^ Department of Pediatrics Emergency Sidra Medicine Doha Qatar; ^2^ Weill Cornell Medical College Doha Qatar; ^3^ Qatar University Medical College Doha Qatar; ^4^ Department of Pediatrics, Division of Pediatric Emergency Hamad Medical Corporation Doha Qatar; ^5^ Retired Seattle Washington USA

**Keywords:** asthma, asthma severity score, length of stay, magnesium intravenous therapy, serum magnesium level, steroid nebulized therapy

## Abstract

**Objective:**

Determine if high‐dose intravenous magnesium with/without nebulized budesonide for children with acute severe asthma is safe and reduces time to medical readiness for discharge compared to standard intravenous magnesium.

**Methods:**

Either 2 doses of intravenous magnesium or 1 dose and placebo, each with/without high‐dose nebulized budesonide, were given in a double‐blind randomized trial to study patients in addition to standard‐of‐care asthma treatment. Time to medical readiness for discharge was the primary outcome. The proportion of patients discharged at 12, 18, and 24 h, asthma severity scores at 4, 8, 12, and 24 h, and safety of high‐dose magnesium were secondary outcomes. Follow‐up was 1 week.

**Results:**

Covid‐19 intervened to stop recuitment at 70% of the estimated 218 children planned. Primary and secondary results were similar for the 4 groups. High‐dose magnesium was safe on clinical and laboratory assessment. Exploratory analysis combining IV high‐dose magnesium groups compared to standard magnesium showed improvement of discharge ratio at 12 h for the former. 40/78 patients (51.2%) in high‐dose magnesium versus 26/75 patients (34.6%) in the standard magnesium arms ready for discharge, *p* = 0.038, absolute risk reduction 16.6%,95% CI, 0.93% to 31.1%. There was a trend toward improvement of PRAM score at 4 and 8 h in the nebulized budesonide groups compared to placebo and no significant difference in seeking post‐discharge outpatient or need for inpatient care.

**Conclusions:**

High‐dose magnesium appeared safe with only a modest possible benefit of medical readiness for discharge at 12 h. There was a reduction in asthma severity score at 4 and 8 h from nebulized budesonide.

**Trial Registration:** clinicaltrials.gov, ID #NCT 02455687

## Key Message

- Single dose of intravenous (IV) magnesium given to children with acute severe asthma has been shown to be safe and described to be effective in reducing hospital admission in multiple previous meta analyses.

- Repeated high doses of intravenous magnesium are safe and effective in reducing discharge rate within 12 h as compared to a single standard dose of intravenous magnesium therapy.

**Abbreviations**
PEC: paediatric emergency center
PICU: paediatric intensive care unit
SD: standard deviation

## 1 Introduction

The prevalence of asthma, reported as 1% to 18% in different countries, shows increasing prevalence in children in recent years [1]. In the USA, asthma prevalence is reported at 7.5% in children < 18 years and 8.6% for school age children [2], with an associated medical cost of childhood asthma treatment estimated to be US$ 4 billion annually [3].

Treatment remains based on pathophysiologic considerations, treatment of bronchospasm and airway inflammation, and relies on delivery of inhaled short‐acting b2 agonists with anticholinergics and systemic corticosteroids [4].

In severe asthma attacks, when patients may not respond adequately to inhaled therapies, intravenous (IV) magnesium and inhaled corticosteroid therapies are tentatively recommended by GINA 2022 guidelines to be considered as additional treatment but with lower evidence levels and a variety of cautions [5].

IV magnesium is posted to help smooth muscle bronchodilation [6]. While there is persuasive evidence in adults that repeated frequent inhaled corticosteroid dosing in the Emergency Department (ED) can reduce need for hospital admission [7], the cost, dose, duration, and which agent(s) are preferred are the caveats cited by GINA experts that dampen the paediatric recommendation. High dose inhaled steroid is posited to work by inducing transient vasoconstriction in the airway mucosa and improving delivery of concomitant inhaled bronchodilator therapy [8].

To evaluate these two approaches, sustained magnesium infusion and early high dose inhaled steroid, placebo‐controlled, alone and in combination, we randomized children with acute severe asthma exacerbations in the paediatric emergency department (ED) to one of four groups. The primary outcome was time to medical readiness for discharge. We hypothesized that a high first dose of intravenous magnesium sulfate (100 mg/kg), followed by a subsequent dose (50 mg/kg) after 4 h, would maintain serum magnesium levels in the recommended therapeutic range (> 2 mmol/L). This regimen was designed to account for the short 2.1‐h half‐life of magnesium in children and the increased renal clearance that occurs during magnesium loading, both of which often limit the sustained efficacy of single standard‐dose infusions.

## 2 Methods

### 2.1 Setting and Participants

The study was conducted between June 2015 and February 2020 in the short stay unit of the Paediatric Emergency Center (PEC) of Hamad General Hospital, one of the main paediatric emergency facilities in the State of Qatar. PEC contains 42 beds in a short stay infirmary unit. Patients admitted to the unit are assessed at least every 6 h by a paediatrician to determine medical readiness for discharge. The length of stay in the unit for asthma has ranged from 3 to 120 h.

Children 2–14 years previously known to have asthma and possessing either nebulized or hand‐held beta‐agonist drug presenting to the unit for treatment of severe asthma exacerbation defined as a PRAM Asthma Severity score ≥ 8 [9] were eligible for the study. Asthma was defined for children between 2 and 5 years as having a previous physician diagnosis of asthma with previous history of eczema in the child or asthma in a first degree relative [10]. For children > 5–14 years, a previous physician diagnosis of asthma was sufficient.

Patients were excluded for one or more of the following characteristics: Preterm birth ≤ 34 weeks gestation, previous history of allergy to magnesium sulfate, presenting with obtundation and progressive respiratory failure requiring ICU admission, hemodynamic instability, history of diagnosis of chronic lung, upper airway disease, congenital heart disease, immunodeficiency or neuromuscular disease, or presence of pneumonic consolidation or pleural disease in chest radiograph at presentation. Written informed consent was sought from one of the parents or legal guardians and assent for children more than 7 years of age for consecutive eligible patients was obtained for all participating patients. The study was approved by Hamad Medical Corporation institutional review board (HMC‐IRB, IRGC‐01‐SI‐006) and registered (clinicaltrials.gov, ID #NCT 02455687).

### 2.2 Study Procedures

This was a blinded randomized placebo‐controlled study. Patients were examined on presentation and those requiring further treatment or observation were admitted to the short stay infirmary unit. Those with an asthma exacerbation were assessed for study eligibility within 15 min of the initial physician assessment. Patients for whom written informed consent was obtained underwent plain chest radiography, nasopharyngeal swab for PCR exam of 12 viruses and IV line insertion. Baseline serum magnesium level was obtained. All enrolled patients were connected to a cardiorespiratory monitor and vital signs were measured every 30 min for at least 6 h after enrollment. A previously computer‐generated list of random numbers was used by the enrolling physicians in consecutive order to identify a sealed envelope, which was accessed and unsealed only by the preparing pharmacist who was blinded to patient identity. The envelopes contained the specific magnesium dosing (high dose or standard dose) and 3 mg budesonide or placebo dosing. A study pharmacist sterilely prepared all intravenous dosages. The high dose IV magnesium arm was prepared as two infusions, the first given as a 100 mg/kg (max 2.5 g) infusion of IV magnesium sulfate, and a second given as a 50 mg/kg (max 2 g) infusion of IV magnesium sulfate given 3 h later if the serum magnesium level was ≤ 2.0 mmol/L. The standard dose IV magnesium arm was prepared as two infusions, the first given as a 50 mg/kg (max 2.5 g) infusion of IV magnesium sulfate, and a second given as an infusion of 0.9% sodium chloride in water given 3 h later if the serum magnesium level was ≤ 2.0 mmol/L. Blinding was preserved through use of clear solutions of the same volumes for all arms. To maximize patient safety, patients were not given the second IV infusion if their serum magnesium level 3 h after the first infusion was above 2 mmol/L or more.

Nebulized budesonide and normal saline placebo were sterilely prepared to deliver 1 mg of budesonide every 20 min for 3 doses, or an equivalent volume of placebo. All randomized patients began their asthma treatment receiving 5 mg of salbutamol and 500 mcg of ipratropium nebulization mixed with 2 mL of normal saline at 0, 20, and 40 min. This was followed by nebulized budesonide or placebo study drug over 20 min, and, if the clinician perceived necessary, a further 5 mg of salbutamol at 60, 80, and 100 min and every 1 h thereafter for 3 doses, then every 2 h until the patient was ready for discharge or admitted. Additional as‐needed nebulized salbutamol 5 mg for 3 doses was allowed to be administered at the discretion of the treating physician. Nebulized therapies were delivered through a tight fitted mask with pressurized 100% oxygen set at 10 L/min. All study patients received IV methylprednisolone 1 mg/kg after enrollment and every 12 h throughout the hospital stay. PRAM Asthma severity score was recorded at baseline, 4, 8, 12, and 24 h unless the patient was discharged earlier. At discharge, patients were sent home with albuterol metered‐dose inhalers with an appropriately‐sized Aerochamber spacer with mask attachment (Forest Laboratories, Dublin, Ireland) and on prednisone, 1 mg/kg once daily (maximum 60 mg) to complete a 5 day course. Study nurse follow‐up by telephone was mandatory daily for 1 week after discharge. The patient could return to the PEC earlier.

### 2.3 Outcome Measures

Time to medical readiness for discharge was the primary outcome measure. Patients were medically ready for discharge when the treating physician determined the patient did not need supplementary oxygen, was feeding adequately without IV fluids, and had minimal or absent wheezing, crackles, or chest retractions, provided the oxygen saturation was ≥ 91% on room air and severity score < 4. Secondary outcomes included asthma severity scores at 4, 8, 12, 48, and 72 h after randomization, proportion of patients discharged within 12, 24, 36, and 48 h post enrollment, need for admission to ICU, and need for revisit or readmission for asthma to any health care facility within 7 days after discharge. To understand pharmacokinetic effect in the high dose magnesium group, blood magnesium level was obtained at 3 and 5 h after first magnesium sulfate infusion for all study patients.

### 2.4 Sample Size and Analysis

An informal review of 10 ED patients with severe asthma during winter showed approximately 50% were medically ready for discharge by 24 h. An improvement of 20%, from 50% to 70% in the high dose magnesium group medically ready for discharge within 24 h would be clinically significant, requiring 109 patients receiving high dose magnesium (half receiving budesonide, half receiving nebulized placebo), 109 receiving standard dose magnesium, for 80% power with two‐sided alpha = 0.05. We intended to enrol 120 high dose magnesium patients, 60 receiving budesonide, 60 receiving placebo, and 120 standard dose magnesium patients, 240 patients altogether, to compensate for dropouts and exclusions.

We used accelerated failure time analysis to compare efficacy for the combined high‐dose magnesium versus standard dose and combined budesonide versus nebulized placebo groups. We also compared medical readiness for discharge at 12, 24, 36, and 48 h, and asthma severity scores at these times. Last, we present individual and mean serum magnesium levels in the treatment groups.

A statistically significant (*p* < 0.05) decrease in the ratio of geometric mean time to medical readiness for discharge would favour a treatment group. Categorical and continuous variables were expressed as percentage and mean with standard deviation (SD). Quantitative variable means between the two treatment groups were analysed by unpaired *t* and Wilcoxon rank sum tests. Associations between qualitative and categorical variables were assessed by chi‐square testing, with the continuity correction for smaller cell frequencies. Statistical analyses were performed using SPSS (version 19.0r, IBM SPSS Statistics, IBM Corporation). Data was transferred from SPSS package to STATA SE 11.0, Chicago, IL for the accelerated time failure analysis.

## 3 Results

Between June 2015 and February 2020, 414 paediatric severe asthma patients were assessed for study eligibility, 160 were randomized after informed consent, and 1 was lost to follow‐up. 78 IV high‐dose magnesium sulfate and 75 standard dose magnesium sulfate patients were available for analysis; patient flow is presented in Figure S1. Recruitment stopped early 2020 due to resource reallocation necessary for Covid‐19. Table 1 presents baseline demographic characteristics of the randomized patients. There were no clinically important baseline differences between the four study groups. The mean baseline asthma severity scores were over 9, mean baseline O2 saturation was 93%, over a third of patients had fever, and 60% had a nasopharyngeal swab positive by PCR testing for one or more viral pathogens. Baseline serum magnesium level was in the normal range and similar for all groups (Table 1).

**TABLE 1:** Baseline characteristics of enrolled patients.

	High dose intravenous magnesium + nebulized budesonide (*N* = 42)	High dose intravenous magnesium + nebulized placebo (*N* = 36)	Standard dose intravenous magnesium + nebulized budesonide (*N* = 34)	Standard dose intravenous magnesium + nebulized placebo (*N* = 41)
Age, years, mean (SD)	5.6 (2.8)	5.4 (2.7)	4.9 (2.8)	5.5 (2.6)
Sex, *n* (%)				
Male	22 (52.4)	22 (61.1)	18 (52.9)	29 (70.7)
Female	20 (47.6)	14 (38.9)	16 (47.1)	12 (29.3)
Baseline asthma severity score, mean (SD)	8.9 (1.0)	9.1 (1.1)	8.9 (0.9)	9.5 (1.1)
Baseline oxygen saturation %, mean (SD)	94.2 (3.6)	92.8 (4.9)	92.9 (4.6)	93.4 (4.3)
Duration of symptoms d, mean (SD)				
Duration of cough	2.2 (1.1)	2.0 (0.8)	1.9 (0.7)	1.8 (0.6)
Duration of difficulty of breathing	1.7 (1.2)	1.4 (0.6)	1.5 (0.7)	1.4 (0.6)
Duration of fever	1.7 (0.8)	1.4 (0.9)	1.5 (0.8)	1.3 (0.5)
Number patients with fever on admission, *n* (%)	16 (38.1)	18 (50.0)	17 (50.0)	20 (48.8)
Number of PEC visits for asthma, preceding year, mean (SD)	4.1 (3.1)	3.8 (2.9)	3.7 (4.7)	2.9 (1.9)
Number of admissions to infirmary for asthma, preceding year, mean (SD)	2.1 (0.9)	2.4 (1.4)	1.8 (1.2)	2.3 (1.2)
Family history of asthma in father, mother or full sibling, *n* (%)	30 (71.4)	23 (63.9)	19 (55.9)	30 (73.2)
History of eczema in patient, *n* (%)	14 (33.3)	10 (27.8)	10 (29.4)	12 (29.3)
Daily exposure to smoking, *n* (%)	5 (11.9)	7 (19.4)	6 (17.6)	6 (14.6)
Daily exposure to pets, *n* (%)	5 (11.9)	3 (8.3)	2 (5.9)	5 (12.2)
Use of prophylactic medication, *n* (%)				
Montelukast	0 (0.0)	3 (8.3)	0 (0.0)	1 (2.4)
Budesonide	1 (2.4)	2 (5.6)	0 (0.0)	5 (12.2)
Fluticasone	4 (9.5)	4 (11.1)	2 (5.9)	4 (9.8)
Fluticasone—salmeterol combination	0 (0.0)	0 (0.0)	0 (0.0)	0 (0.0)
Chest radiograph, *n* (%)				
Normal	16 (38.1)	11 (30.6)	7 (21.2)	13 (32.5)
Atelectasis	2 (4.8)	7 (19.4)	4 (12.1)	6 (15.0)
Increased bronchovascular markings	21 (50.0)	17 (47.2)	21 (63.6)	19 (47.5)
Pneumonia with consolidation	2 (4.8)	1 (2.8)	1 (3.0)	1 (2.5)
Fibrotic changes	1 (2.4)	0 (0.0)	0 (0.0)	0 (0.0)
Positive PCR in nasopharyngeal swab, *n* (%)	30 (71.4)	32 (88.9)	26 (76.5)	29 (70.7)
Rhinovirus positivity in PCR nasopharyngeal swab, *n* (%)	20 (48.8)	20 (55.6)	16 (47.1)	14 (34.1)
Enterovirus positivity in PCR nasopharyngeal swab, *n* (%)	0 (0.0)	1 (2.8)	1 (2.9)	2 (4.9)
RSV positivity in PCR nasopharyngeal swab, *n* (%)	1 (2.4)	0 (0.0)	3 (8.8)	0 (0.0)
Influenza/parainfluenza viruses positivity in PCR nasopharyngeal swab, *n* (%)	1 (2.4)	1 (2.8)	0 (0.0)	2 (4.9)
Bocavirus positivity in PCR nasopharyngeal swab, *n* (%)	0 (0.0)	2 (5.6)	0 (0.0)	1 (2.4)
Adenovirus positivity in PCR nasopharyngeal swab, *n* (%)	2 (4.8)	2 (5.6)	1 (2.9)	1 (2.4)
Coronavirus positivity in PCR nasopharyngeal swab, *n* (%)	0 (0.0)	2 (5.6)	1 (2.9)	1 (2.4)
Human metapneumovirus positivity in PCR nasopharyngeal swab, *n* (%)	0 (0.0)	0 (0.0)	0 (0.0)	0 (0.0)
Mycoplasma positivity in PCR nasopharyngeal swab, *n* (%)	0 (0.0)	0 (0.0)	0 (0.0)	0 (0.0)
Co‐infection with more than 1 organism in PCR nasopharyngeal swab, *n* (%)	6 (14.3)	6 (16.7)	4 (11.8)	7 (17.1)
Basal magnesium level (mmol/L), mean (SD)	0.81 (0.07)	0.81 (0.07)	0.83 (0.09)	0.83 (0.17)

Blood magnesium levels at 3 h after first magnesium sulfate infusion, obtained for all study patients in each group, were mean (SD) 1.20 (SD 0.12) mmol/l (high‐dose plus budesonide) versus 1.18 (SD 0.129) (high‐dose plus placebo) versus 0.96 (SD 0.06) (standard dose plus budesonide) versus 0.97 (SD 0.06) (standard dose plus placebo), (*p* = 0.000, Student *t* test) and at 5 h 1.56 (SD 0.26) versus 1.52 (SD 0.31) versus 0.96 (SD 0.06) versus 0.97 (SD 0.06), (*p* = 0.000, Student *t* test) for the same groups, respectively. All patients received the second magnesium/placebo dose, Figure 1 shows individual levels over time.

**FIGURE 1:**
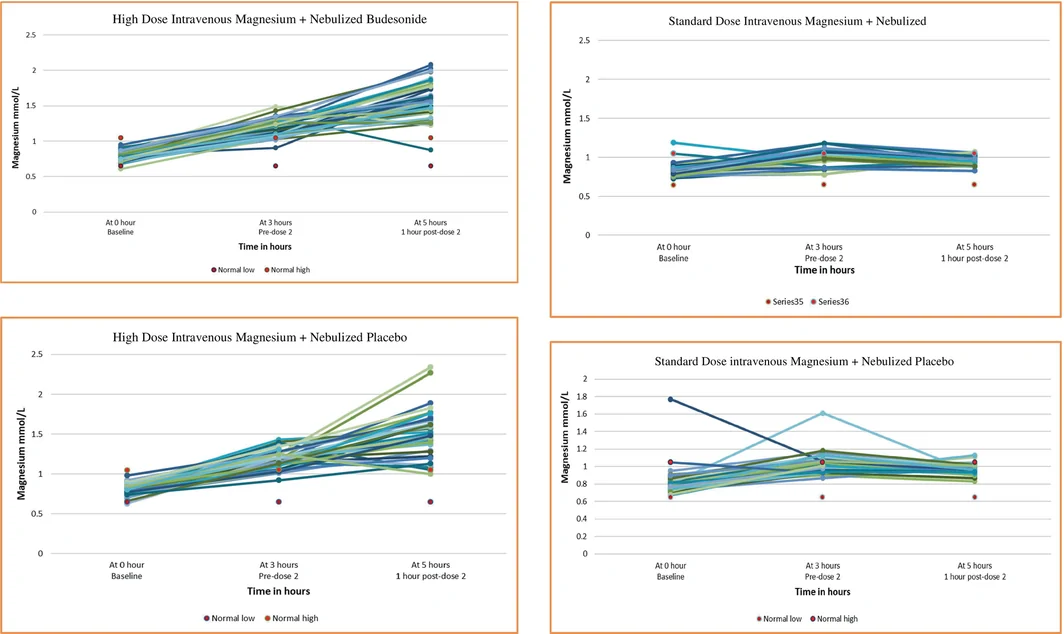
Serum magnesium level at 3‐time intervals. Serum Magnesium level at 3‐time interval: 0 h: Right before IV magnesium infusion. 3 h: 3 h after 1st magnesium infusion. 5 h: 5 h after 1st magnesium infusion.

The geometric mean time to readiness for discharge was 13.4 h (95% CI 11.46 to 15.68) for the high magnesium with nebulized budosenide group, 13.16 h (95% CI 11.08 to 15.62) for the high magnesium with nebulized placebo groupo, 14.30 h (95% CI 12.24 to 16.70) for the standard magnesium with nebulized budesonide group, and 13.38 h (95% CI 11.36 to 15.76) for the standard magnesium with nebulized placebo group, *p* = 0.718 (Figure 2). There were no significant differences between the four groups. Asthma severity scores at 4 and 8 h were numerically lower in the high magnesium group with budesonide when compared with other groups (Table 2). Exploratory analysis combining the IV high dose magnesium groups and comparing to standard magnesium dosing combined groups, for medical readiness for discharge and other secondary outcomes, showed no difference (Figure S2A, Table 3), except for discharge by 12 h, 40/78 patients (51.2%) in high dose IV magnesium sulfate versus 26/75 patients (34.6%) in the standard magnesium arms ready for discharge, *p* = 0.038, absolute risk reduction for longer than 12 h, 16.6%, 95% CI, 0.925% to 31.14%.

**FIGURE 2:**
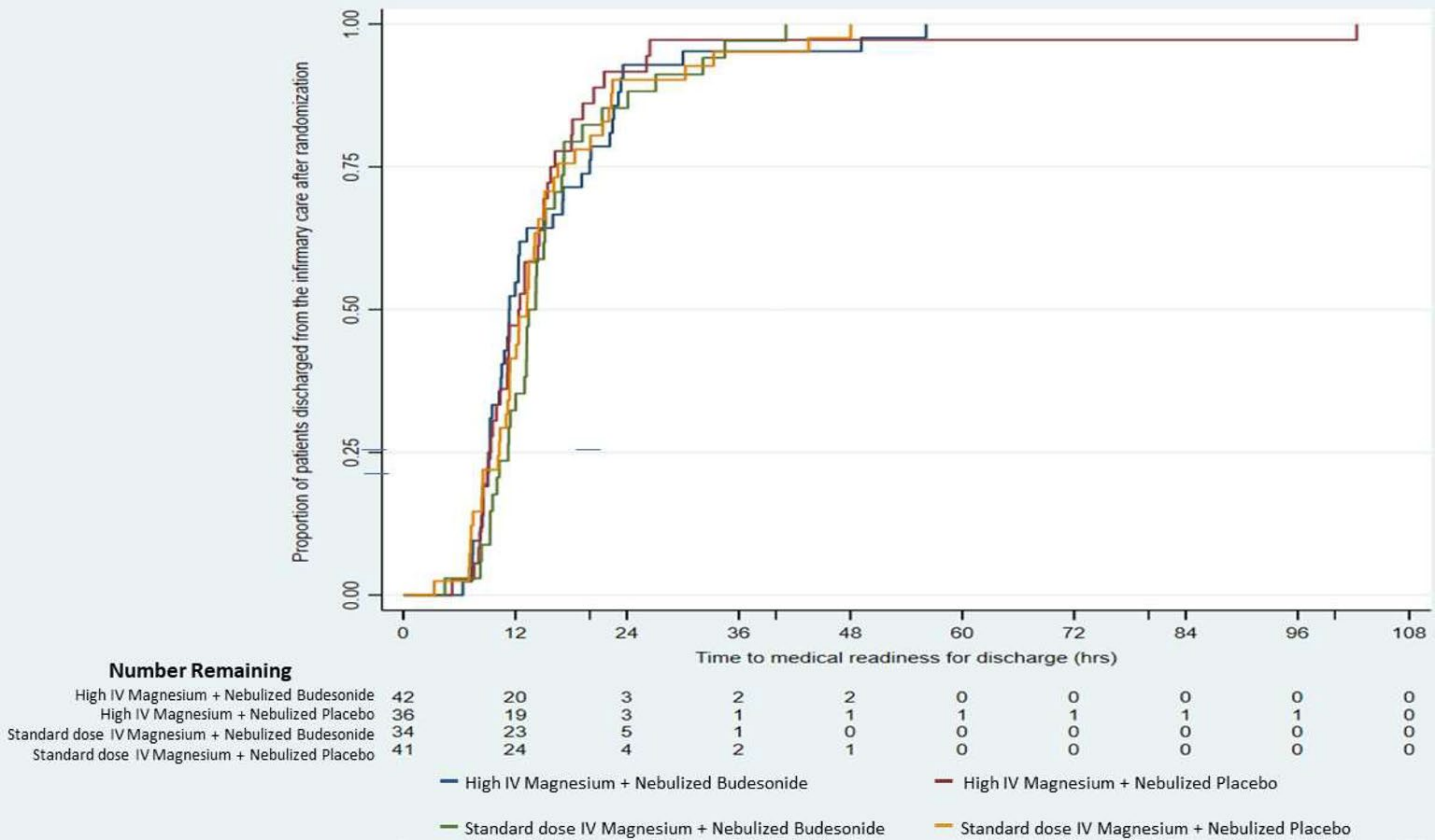
Time to medical readiness for discharge for the 4 study groups.

**TABLE 2:** Outcome: primary and secondary outcome measures for the 4 study groups.

	High dose intravenous magnesium + nebulized budesonide (*N* = 42)	High dose intravenous magnesium + nebulized placebo (*N* = 36)	Standard dose intravenous magnesium + nebulized budesonide (*N* = 34)	Standard dose intravenous magnesium + nebulized placebo (*N* = 41)	*p*
Primary outcome					
Time to medical readiness for discharge, mean (SD)	15.5 (10.3)	15.8 (15.7)	15.8 (7.9)	15.4 (9.3)	1.0
Secondary outcome					
Asthma severity score, mean (SD), *p* value					
Baseline asthma severity score	8.9 (1.0)	9.1 (1.1)	8.9 (0.9)	9.5 (1.1)	0.08
Asthma severity score after 4 h	4.9 (1.4)	5.9 (1.8)	5.3 (1.4)	5.9 (2.0)	0.01
Asthma severity score after 8 h	3.9 (1.3)	4.9 (1.9)	4.1 (1.34)	4.3 (1.7)	0.08
Asthma severity score after 12 h	3.84 (1.42)	4.00 (1.73)	4.38 (1.26)	4.00 (1.68)	0.8
Asthma severity score after 24 h	6.7 (2.9)	3.4 (0.5)	3.7 (1.9)	5.2 (1.5)	0.09
Asthma severity score on discharge	2.6 (0.5)	2.4 (0.8)	2.8 (0.6)	2.5 (1.0)	0.1
No. of patients requiring PICU admission. *n* (%), *p* value	0	1 (2.8)	0	0	0.3
Revisit to any healthcare facility within 1 week of follow‐up, *n* (%), *p* value	3 (7.1)	2 (5.7)	5 (14.7)	3 (7.3)	0.5
Readmission to any healthcare facility within 1 week of follow‐up, *n* (%), *p* value	1 (2.4)	1 (2.9)	1 (2.9)	0	0.8
Serum Magnesium level, mean (SD), *p* value					
Serum Magnesium level at 3 h after enrolment	1.20 (0.12)	1.18 (0.12)	1.00 (0.11)	1.04 (0.12)	0.00
Serum Magnesium level at 5 h after enrolment	1.56 (0.26)	1.52 (0.31)	0.96 (0.06)	0.97 (0.06)	0.00

**TABLE 3:** Primary and secondary outcome measures for high dose magnesium vs. standard dose magnesium and for nebulized budesonide vs. nebulized placebo.

	High dose intravenous magnesium + nebulized budesonide and high dose intravenous magnesium + nebulized placebo (*N* = 78)	Standard dose intravenous magnesium + nebulized budesonide and standard dose intravenous magnesium + nebulized placebo (*N* = 75)	*p*	High dose intravenous magnesium + nebulized budesonide and standard dose intravenous magnesium + nebulized budesonide (*N* = 76)	High dose intravenous magnesium + nebulized placebo and standard dose intravenous magnesium + nebulized placebo (*N* = 77)	*p*
Primary outcome						
Time to medical readiness for discharge, mean (SD)	13.3 h (11.9 to 14.9 h)	13.8 h (12.3 to 15.4 h)	0.3	13.8 h (12.4 to 15.4 h)	13.3 h (11.8 to 14.9 h)	0.6
Ratio of geometric means	1.1 h (0.9 to 1.2 h)			1.1 h (0.9 to 1.2 h)		
Secondary outcome						
Asthma severity score, mean (SD), *p* value						
Baseline asthma severity score	9.0 ± 1.0	9.2 ± 1.0	0.2	8.9 ± 0.9	9.3 ± 1.1	0.08
Asthma severity score after 4 h	5.3 ± 1.7	5.7 ± 1.8	0.3	5.0 ± 1.4	5.9 ± 1.9	0.00
Asthma severity score after 8 h	4.4 ± 1.7	4.2 ± 1.6	0.6	3.9 ± 1.3	4.6 ± 1.8	0.03
Asthma severity score after 12 h	3.9 ± 1.5	4.2 ± 1.5	0.5	4.1 ± 1.4	4.0 ± 1.7	0.8
Asthma severity score after 24 h	4.6 ± 2.3	4.5 1.8	0.9	5.0 ± 2.6	4.2 ± 1.4	0.5
Asthma severity score on discharge	2.5 ± 0.7	2.6 ± 0.8	0.3	2.7 ± 0.6	2.4 ± 0.9	0.04
No. of patients requiring PICU admission. *n* (%), *p* value	1 (1.3%)	0 (0%)	1.0	1 (1.3%)	0 (0%)	1.0
Revisit to any healthcare facility within 1 week of follow‐up, *n* (%), *p* value	5 (6.5%)	8 (10.7%)	0.35	8 (10.5%)	5 (6.6%)	0.38
Readmission to any healthcare facility within 1 week of follow‐up, *n* (%), *p* value	2 (2.6%)	1 (1.3%)	1.0	2 (2.6%)	1 (1.3%)	1.0

When combining the nebulized budosenide groups and comparing to nebulized placebo for medical readiniess for discharge and other outcomes (Figure S2B, Table 3), the only significant finding was the mean asthma severity score at 4 and 8 h in the combined budosenide groups was less than the mean in the placebo groups, exceeding the baseline difference.

### 3.1 Safety

Vital signs at observation times were similar between the treatment groups. No hypotension was observed. Two patients in the high dose magnesium group had facial flushing after the first infusion but were not removed from the study. One patient from the high dose magnesium with budesonide group required paediatric ICU admission for refractory status asthmaticus.

### 3.2 Revisits and Re‐Admissions

The frequency of outpatient re‐visits to any medical facility for the same illness in the 7 days after discharge was: 5/78 (6.4%) in the high dose magnesium and 8/75 (10.6%) in the standard magnesium groups, *p* = 0.35. For the nebulized budesonide and placebo groups, the frequencies were 8/76 (10.5%) and 5/77 (6.5%), *p* = 0.38. Re‐admission to hospital or infirmary care was needed for 2/78 (2.5%) in the high dose magnesium and 1/75 (1.3%) in the standard magnesium groups, *p* = 1.0. For the budesonide groups, the rates were 2/76 (2.6%) and 1/77 (1.3%) respectively, *p* = 1.0.

## 4 Discussion

Our trial, stopped prematurely due to Covid‐19 taking resource priority with but 70% of our estimated sample size, showed only improvement of discharge within 12 h for the combined high magnesium groups as compared to the standard magnesium groups (Figure S2A), a reduced asthma severity score at 4 and 8 h for the combined inhaled budesonide groups, and did not show clinical safety concerns from beginning magnesium therapy with a high dose and following 4 h later with a standard dose.

The data for magnesium appears ambiguous. In acute severe asthma, magnesium may have several possible beneficial effects, including decreasing intracellular calcium by blocking its entry to the cell, blocking its release from the endoplasmic reticulum, and activating sodium‐calcium pumps. It appears to inhibit the interaction btween calcium and myosin reducing bronchial muscle fibre excitability, stabilizes T cells, inhibits mast cell degranulation, and stimulates nitric oxide and prostacyclin synthesis [11, 12, 13]. Athough nebulized magnesium added to usual paediatric emergency department treatment does not reduce the need for admission [14, 15], the evidence for intravenous (IV) magnesium ramains more nuanced. While earlier meta‐analyses based on three randomized clinical trials [16, 17, 18] described IV magnesium as effective in reducing hospital admissions, these fundamental studies were conducted prior to routine use of concurrent ipratropium in standard paediatric emergency care. Recent systematic reviews have recognised that the antiquated nature of these trials, which lacked the modern standard of care context, limits their applicability to current practice. This may explain why IV magnesium was not associated with improved hospital outcomes in a recent retrospective study of patients in non‐ICU beds [19] or in secondary analyses of refractory patients. Keep in mind the three RCTs mentioned were all conducted prior to the development of current used paediatric emergency department treatment, with no concurrent use of ipratropium [20]. When given at physician discretion to paediatric asthmatics failing other therapies, IV magnesium was associated with a higher likelihood of admission in a subgroup secondary analysis [21]. In the aforementioned studies of IV magnesium, the duration of infusion was either 30 min or not described. IV magnesium has an average half‐life of 2.1 h in children [22]. Maintaining a magnesium level above 2 mmol/L is recommended to induce smooth muscle relaxation in acute asthma [23]. Renal magnesium clearance rises as serum magnesium increases above a normal level [24], making it challenging to achieve and sustain a therapeutic level with a single time‐limited infusion.

We sought to determine if front‐loading a high magnesium dose would allow children with severe asthma to be discharged sooner than standard treatment. As a secondary outcome, we sought to determine if we might prevent ICU admissions for our participants. At our center, in addition to some other centres, children with refractory asthma admitted to the ICU after ED treatment are begun on a continuous magnesium infusion as part of their ongoing treatment. Because we measured magnesium blood levels at baseline and before the second dose and 1 h after the second dose, we are able to present new emergency department data showing the safety of beginning magnesium treatment with a high dose. With our reduced patient number due to Covid‐19, our hypothesis could not be proven or disproven.

We found no harm but some apparent benefit for a single high dose of nebulized budesonide. Since this approach to budesonide administration offered some benefit, it remains possible that repeated high doses of nebulized budesonide, as has been reported beneficial in asthmatic adults [25, 26], in combination with high‐dose magnesium might offer benefit, but further studies would be required to investigate this.

Our study has important limitations. Eligibility was determined by high PRAM severity score for asthma, rather than by a patient being refractory to usual treatment, the usual circumstance that prompts magnesium infusion. We chose this approach because relatively few patients are refractory and the judgement of being refractory can be criticized as subjective. For budesonide, a single nebulization might not be sufficient, since success in adults was achieved with multiple inhaled steroid doses. Our unplanned reduced sample size is an important limitation; it could have prevented the opportunity to show further benefit in the high‐dose magnesium and nebulized budesonide groups.

In the context of our Covid‐19 reduced number of recruited patients, we conclude that administering high dose magnesium to children presenting to the ED with severe asthma attacks by PRAM score appeared safe clinically and by measured blood level but we identified only modest benefit of medical readiness for discharge within 12 h, and also reduction in asthma severity score at 4 and 8 h from the single nebulized budesonide dose.

## Author Contributions

Khalid Alansari, Hadi Abu Rasheed, Fatin Abou Moussa, and Bruce L. Davidson did the literature search, study design, data analysis and interpretation, and primary drafting of the manuscript. Khalid Yousif Ibrahim and Mahmoud Alrifaai were involved with Khalid Alansari and Fatin Abou Moussa in patient recruitment. Each of the six authors contributed to manuscript revisions and important intellectual content and approved the final manuscript as submitted.

## Funding

This study was hospital‐sponsored by Hamad Medical Corporation with a grant of US $250 000.

## Conflicts of Interest

The authors declare no conflicts of interest.

## Supporting information


**Figure S1:** Consort form of patients flow.

**Figure S2A:** Time to medical readiness for discharge for all patients who received high dose.
**Figure S2B:** Time to medical readiness for discharge for all patients who received nebulized.

## Data Availability

The data that support the findings of this study are available on request from the corresponding author. The data are not publicly available due to privacy or ethical restrictions.
