# Supplementary material for: A Pilot Placebo‐Controlled Randomized Trial of Repeated High‐Dose Intravenous Magnesium Sulphate and High‐Dose Nebulized Budesonide for Severe Asthma in the Paediatric Emergency Department
Source: J Paediatr Child Health. 2026 Aug 16;62(10):1948–58. doi: 10.1111/jpc.70530 (PMC13671247; doi:10.1111/jpc.70530)

Supplemental Figure 1: Consort Form of patients flow

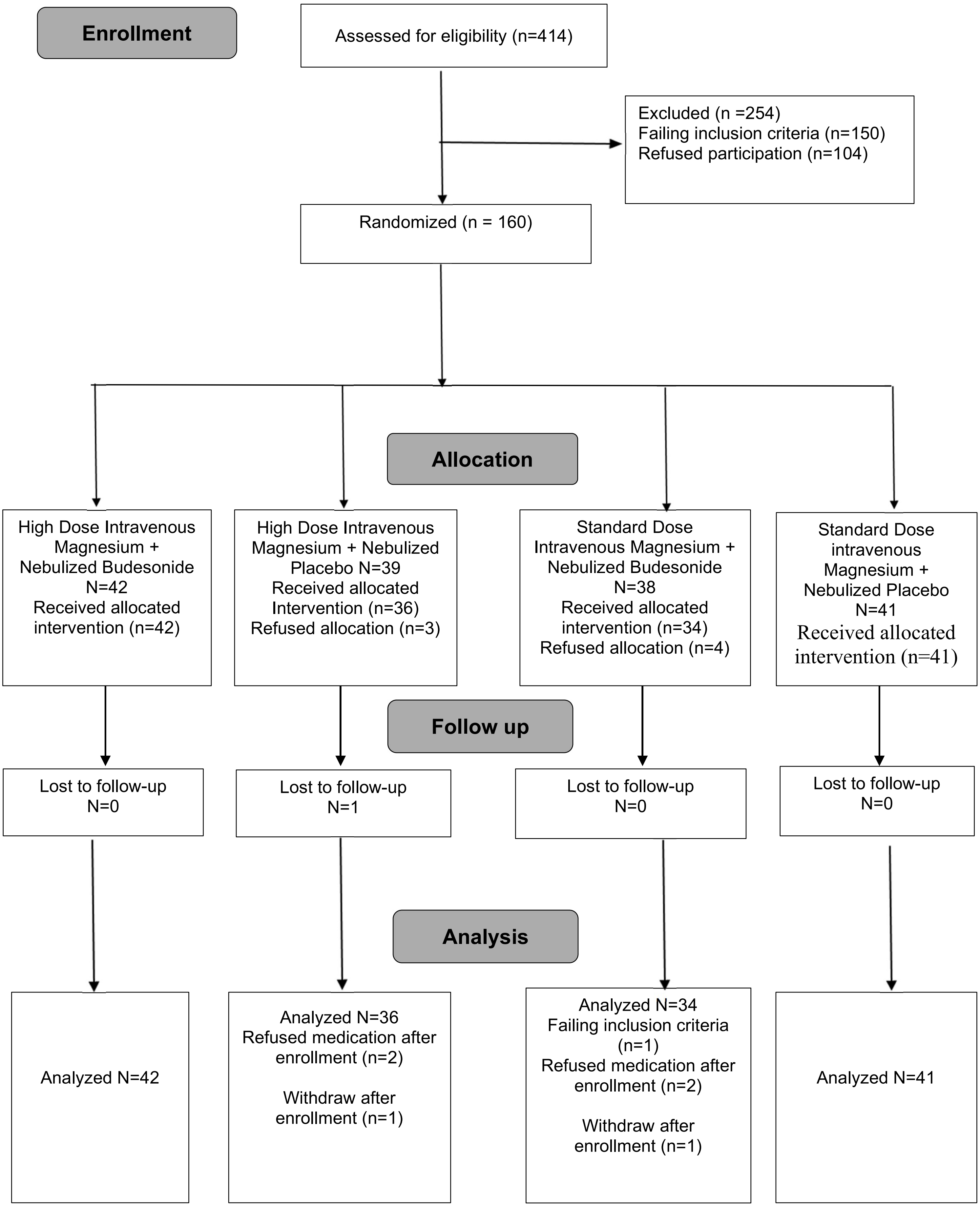

Supplement: Supplementary file 1 [file JPC-62-1948-s001.pdf]
