# Supplementary material for: A Pilot Placebo‐Controlled Randomized Trial of Repeated High‐Dose Intravenous Magnesium Sulphate and High‐Dose Nebulized Budesonide for Severe Asthma in the Paediatric Emergency Department
Source: J Paediatr Child Health. 2026 Aug 16;62(10):1948–58. doi: 10.1111/jpc.70530 (PMC13671247; doi:10.1111/jpc.70530)

Supplemental Figure 2A: Time to medical readiness for discharge for all patients who received high dose

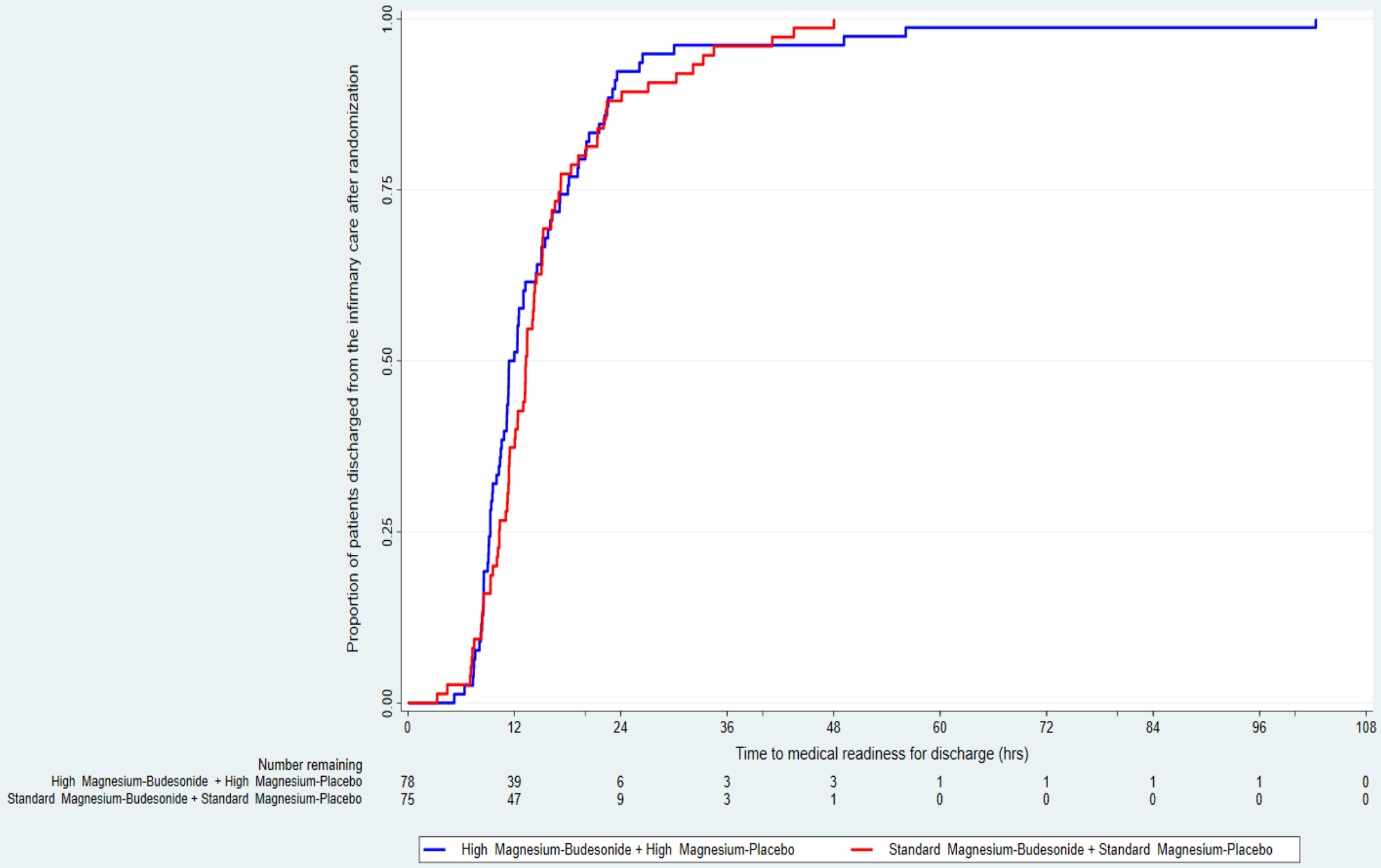

Supplemental Figure 2B: Time to medical readiness for discharge for all patients who received nebulized

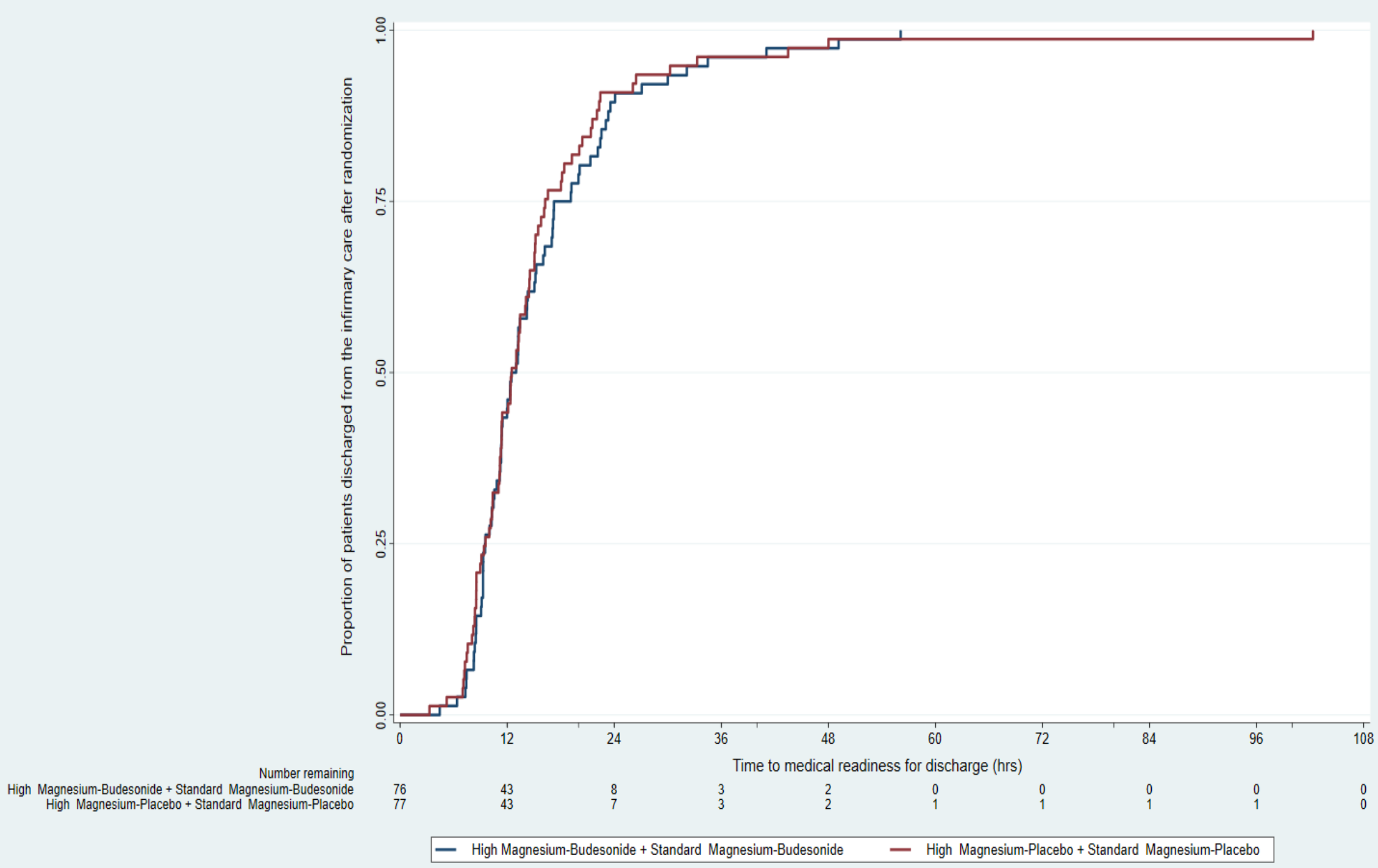

Supplement: Supplementary file 2 [file JPC-62-1948-s002.pdf]
